# Sub-optimal femoral fit in total knee arthroplasty, a systematic review of human femoral data vs off-the-shelf contemporary femoral components

**DOI:** 10.1186/s40634-023-00607-x

**Published:** 2023-04-10

**Authors:** Tommaso Bonanzinga, Francesco Manlio Gambaro, Francesco Iacono, Maurilio Marcacci

**Affiliations:** 1 IRCCS Humanitas Research Hospital, via Manzoni 56, 20089 Rozzano, Milan, Italy; 2 Department of Biomedical Sciences, Humanitas University, Via Rita Levi Montalcini 4, 20090 Pieve Emanuele, Milan, Italy

**Keywords:** Knee, TKA, Morphological analysis, Reverse engineering

## Abstract

**Purpose:**

The purpose of the current study is to investigate the inadequacy of fit between the human distal femur and the knee implants offerings and describe the available strategies to overcome this issue.

**Methods:**

A systematic research of the literature was performed to identify studies reporting morphologic measures of the distal femur. Studies were excluded if they included unhealthy knees or the morphological analysis did not report the two key dimensions to identify the patient’s unique anatomy: AP length and mediolateral (ML) width. Clinically relevant component overhang or underhang was considered when the metal-bone mismatch was > 3 mm as described in the literature.

**Results:**

Six studies with anthropometric analysis of 1395 distal femurs met the inclusion criteria. The analysis revealed that by employing the available sizes of four current “state-of-the-art” primary off-the shelf (OTS) femoral implants up to 13–41% would show underhang and 9–27% overhang clinically relevant and the introduction of narrower sizes did not reduce this percentage of underhang but improved the overhang rate of 10–15%.

**Conclusions:**

Whenever an ML/AP mismatch in encountered in the operating room, adaptations are needed, and these bring about deleterious biomechanical and clinical complications. Therefore, this study highlights the need for implants design with multiple ML offerings per AP size, since they provide not only more sizes options but more femoral shapes to match the different ML sizes of the distal femur, compared to designs with single ML offerings for a given femoral AP dimension.

## Introduction

Femoral component mismatch with respect to the native bone morphology represents a key challenge in TKA and the purpose of the current study is to investigate its frequency and discuss possible solutions.

The outcomes of total knee arthroplasty (TKA) look very different based on the parameters employed to analyze them. Indeed, when looking at the revision rate, knee implants exhibit excellent results with a 10 years revision rate estimated at 5%, that grows to 6,7% at 15 years and 8,0% at 19 years [8]. Nevertheless, when moving to patient reported outcomes measures (PROM), modern TKA implants performance drops considerably. Indeed, patients’ dissatisfaction, mainly related to residual knee pain and knee function abnormalities, is estimated as high as 20% with a higher incidence in the female population [11]. The reasons behind this observed dissatisfaction are heterogenous and can be classified into patient-specific or surgery-specific. The patient-specific factors vary from sociodemographic (worse outcomes in African Americans patients [2] and females [20]) to preoperative (unmet expectations, delay in surgery, poor preoperative PROM [4]) and postoperative factors (longer hospital stay, low activity level [11]). Among the surgery specific factors should be mentioned the surgical technique and the implant design. The influence of the latter on the abovementioned post-operative patients’ dissatisfaction is the focus of our study. Indeed, the current TKA prosthetic design is based on a single shape implant with few sizes designed based on the average morphology of the white Caucasian male knee, therefore predisposing to possible mismatches when employed in the knee shape heterogeneity seen in the clinical practice.

This inadequacy of fit may arise from one of the different components of the implant: tibial, femoral and patellar in case of patellar resurfacing. Concerning the femoral component, the inadequacy of fit has been studied in the literature by looking at the discrepancy between the anteroposterior (AP) and mediolateral (ML) sizes on the axial plane of the distal femur of the patient compared with these same axes of the prosthesis implant. Indeed, Mahoney et al. [17] found that an overhang of at least 3 mm of the metal beyond the bone cut on the ML axis edge was present in 68% of women and 40% of men, confirming the negative trend in the female population. They also observed that femoral component overhang of > 3 mm in at least one zone was associated with an almost twofold increased risk of knee pain more severe than occasional or mild at two years after surgery (odds ratio: 1.9). The pathophysiological basis behind the higher rate of residual pain observed may be caused by the fact that soft tissue impingement due to TKA component overhang can lead to osteophytes formation, extruded bone cement and irritations of surrounding ligaments and tendons [10]. These results were confirmed also by other studies who found that femoral implants ML oversizing is frequent (68%), especially in women and correlated with worse KOOS score and a lower ROM in flexion [3, 14]. This high frequency of incorrect femoral implant sizing likely stems from the discrepancy between a well-known high variability in the size and shape of the distal femur and the limited ML/AP offerings of the current off the shelf (OTS), available implants [9].

The purpose of the current study is to investigate the frequency of the suboptimal fit on the femoral ML and AP axes.

## Materials and methods

A systematic review was conducted and finalized on May 1^st^, 2022 according to the Preferred.

**Fig. 1:**
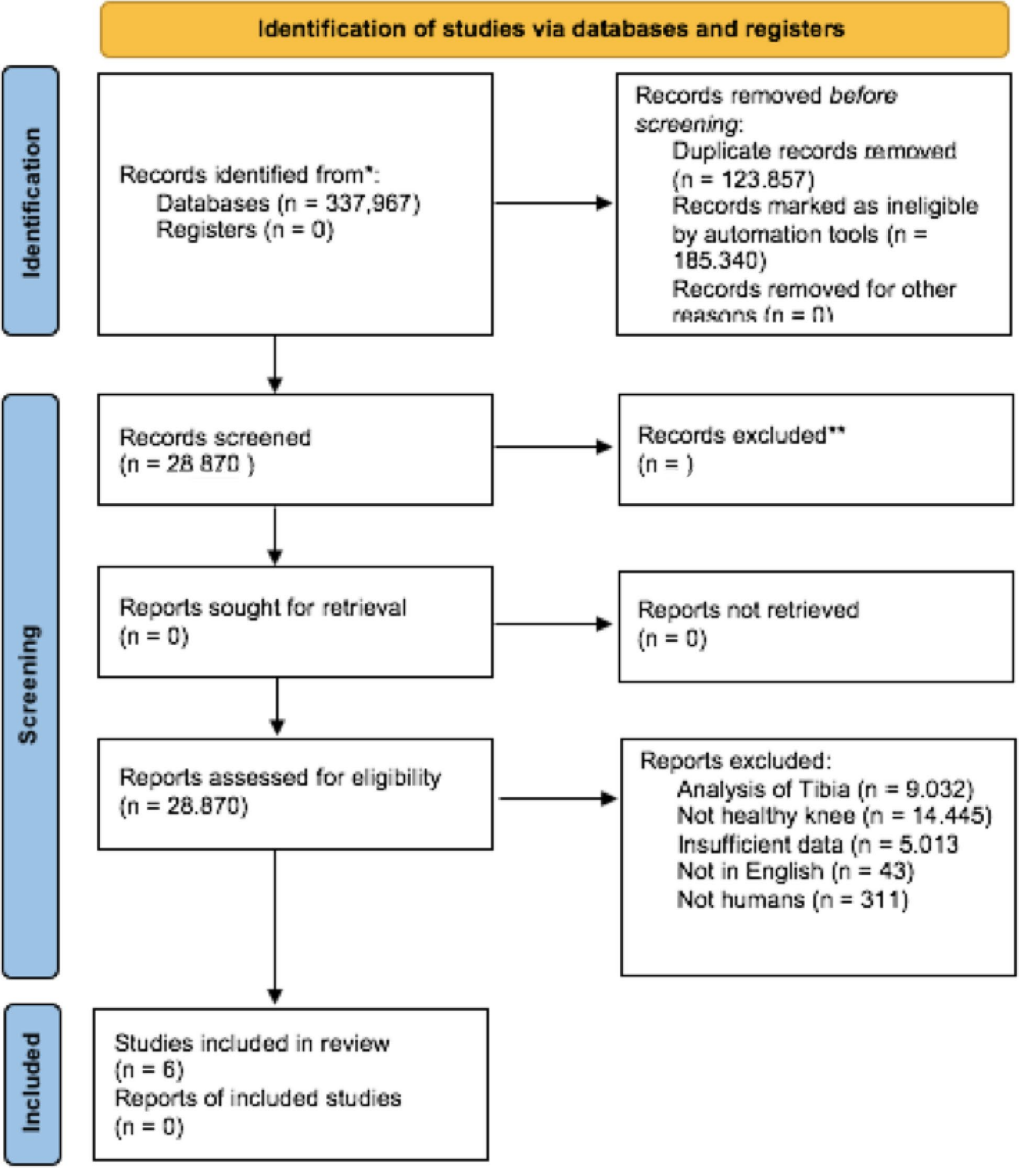
Flowchart of the Preferred Reporting Items for Systematic Reviews and Meta-Analyses (PRISMA)

Reporting Items for Systematic Reviews and Meta-Analyses (PRISMA) as depicted in Fig. 1 [21]. A systematic research was performed on the PubMed and Google Scholar databases and were included studies reporting morphologic measures of the distal femur, written in English, published between the dates January 2000 and October 2021 using a combination of the following words: “Morphological”, “Anthropometric”, “Femur”, “Femoral”, “Distal femur”, “Knee”. Conversely, studies were excluded if they were not written in English and if they investigated also unicompartmental knee arthroplasty or trauma products, exclusively measured the patella, were conducted in pediatric or nonhuman subjects, featured data unrelated to the distal femur, or data that were considered insufficient for analysis. Included studies were also assessed to confirm there were no duplicate patient cohorts. Each included article was assessed through the National Institutes of Health (NIH) Quality Assessment Tool for Observational Cohort and Cross-Sectional Studies and rated accordingly as “good”, “fair” or “poor”.

**Fig. 2:**
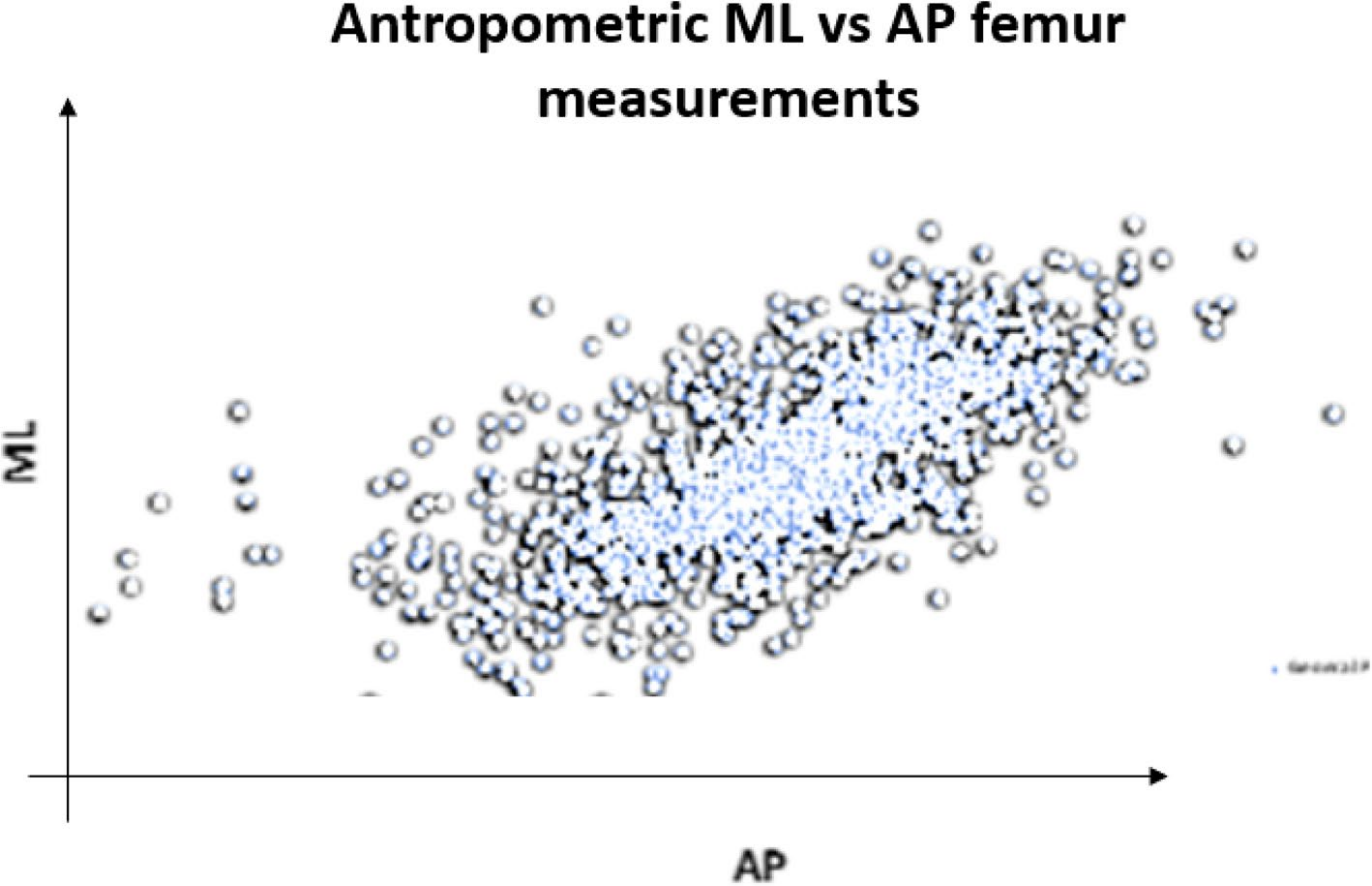
Visual representation of the dataset, where each dot represents a femur described by a couple of coordinates (AP, ML)

The distal femoral morphological data in terms of ML and AP sizes were extracted from each case of each included article and by reverse engineering all the datapoints were plotted on a scatterplot (Fig. 2).

**Fig. 3:**
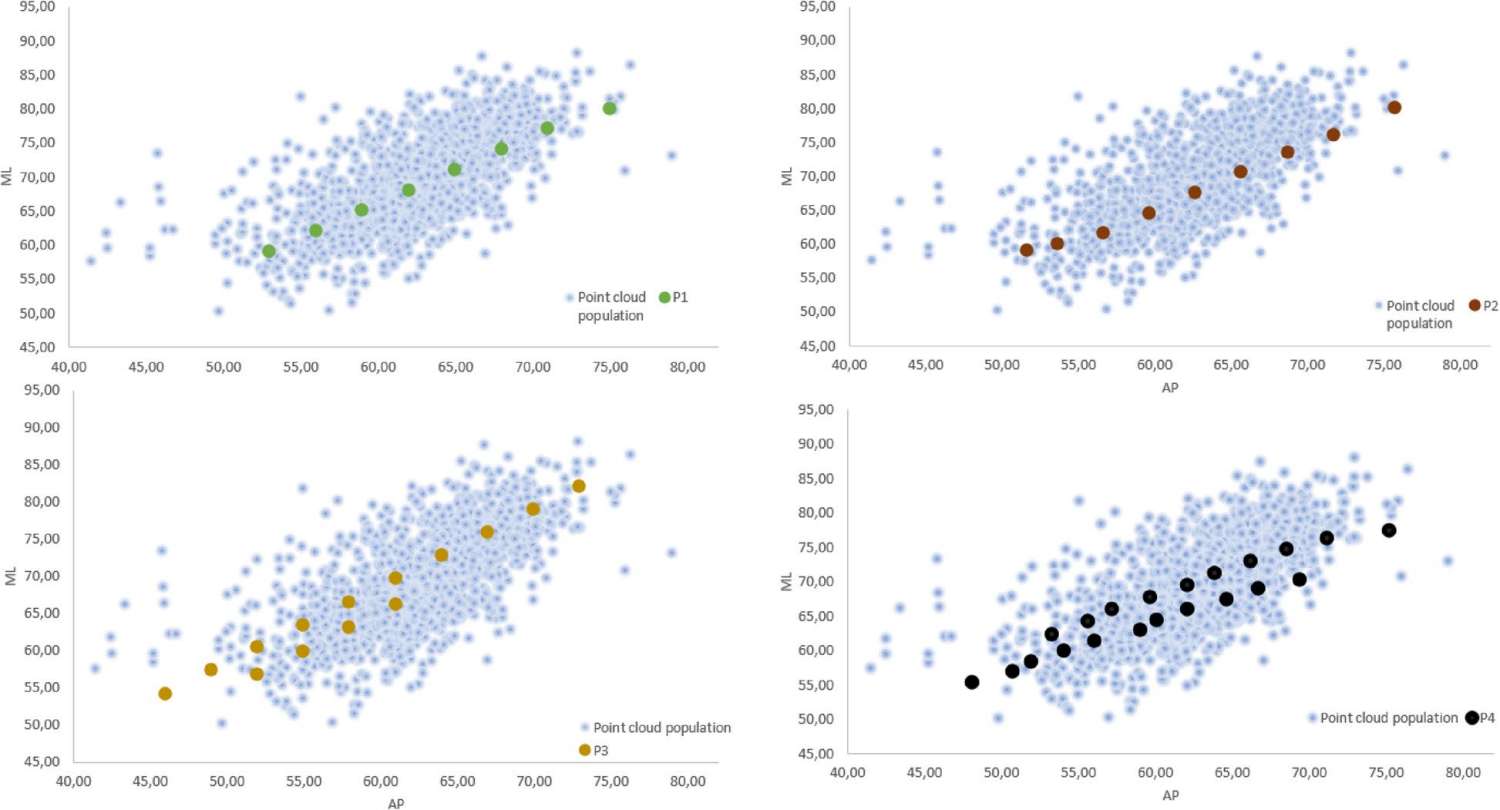
Comparison of the ML and AP dimensions of the population datapoints and OFT implants

The population datapoints, representing the anatomical dimensions of the femur, were then compared with the ML and AP dimensions of 4 off-the-shelf (OTS) commonly used TKR (Total Knee Replacement) system, here referred as P1, P2, P3 and P4. The TKR implants were all CR (Cruciate Retaining) versions, and the ML and AP dimensions are reported in Fig. 3.

According to the definition of overhang and underhang [17], a perfect fit scenario falls, for a given AP, within a tolerance of +—3 mm for ML.

**Fig. 4:**
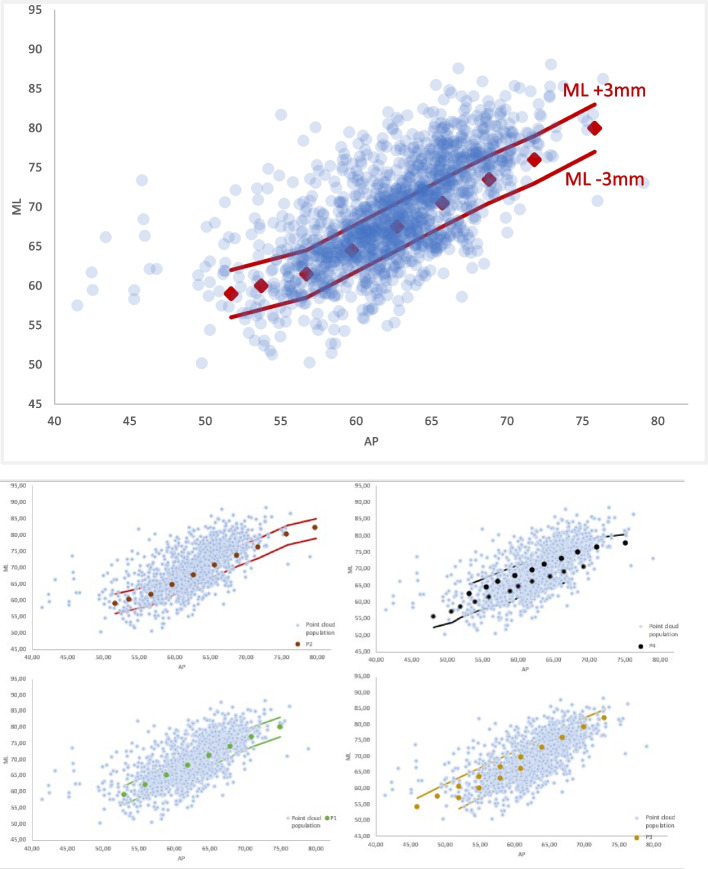
For each OTS there is a line representing the average and parallel lines for ML + -3 mm were considered

In this study, for each OTS device, a line was drawn through the size dimension and parallel lines for ML + -3 mm were considered, as shown in Fig. 4. The two lines includes all the population datapoints which would benefit of a best-fit implant, while the population with anatomical dimensions above the ML + 3 mm line would receive not best-fit implants, with smaller ML dimensions, potentially causing underhang. Conversely, the population with anatomical dimensions below the ML -3 mm line, with smaller ML dimensions than the available implants, would incur in overhang. The number of datapoints below and above the two lines were evaluated and for each OTS device and reported as percentage of the whole population.

## Results

The search identified 337,967 potentially eligible published studies. After review of the title, abstract, and full text by one of the authors (MP), 337.961 of these studies were excluded and at final screening only 6 articles [6, 7, 15, 16, 18, 19] met the inclusion criteria and 1395 femoral measurements were finally included in the current study. A description of patient’s demographic included in the finally selected articles is presented in Table 1.

**Table 1:** Presents a summary of the included articles

Article	Population	Knees	Method	Endpoint	NIH ass	Reference
Chaichankul 2011	Thailand	200	MRI	AP and ML	Fair	[13]
Cheng 2009	Chinese	172	CT	AP and ML	Fair	[14]
Li 2014	Caucasian	275	MRI	AP and ML	Good	[15]
Magetsari 2015	Indonesian	100	CT	AP and ML	Fair	[16]
McNamara 2018	Hispanic	500	MRI	AP and ML	Fair	[17]
Moghtadaei 2014	Iranian	150	CT	AP and ML	Fair	[18]

Legend: *CT* Computed-Tomography, *MRI* Magnetic Resonance Imaging, *AP* Antero-Posterior, *ML* Medio-lateral

**Fig. 5:**
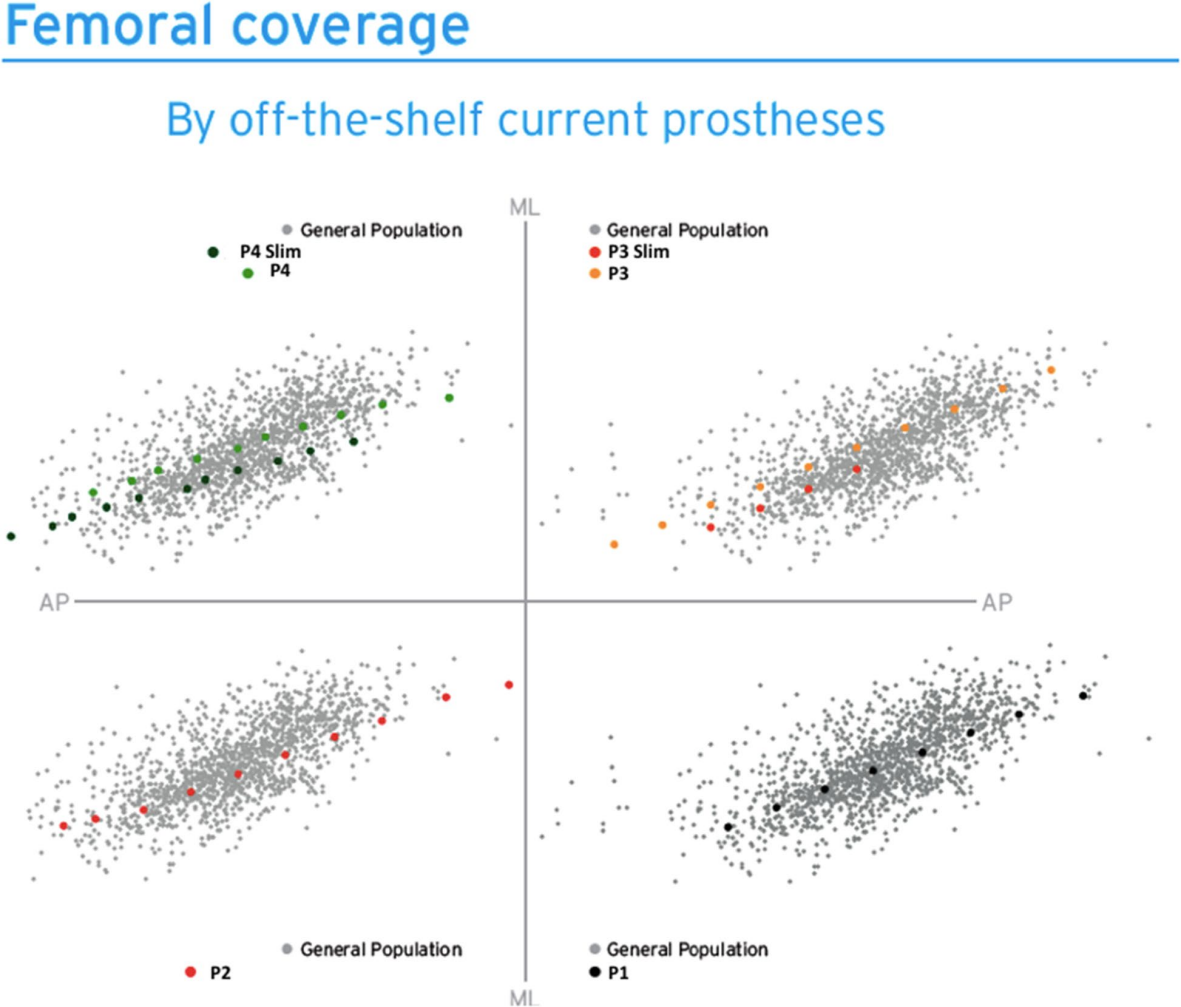
Coverage offered by off the shelf traditional femoral prostheses (P1, P2, P3, P4 and the Slim version of P3 and P4). Each grey dot depicts an individual femur in the database and each colored dot a femoral implant size. Legend: ML medio-lateral, AP: antero-posterior

The technique of reverse plot digitalization on the anthropometric data of the included studies generated the graph depicted below in Fig. 5.

**Fig. 6:**
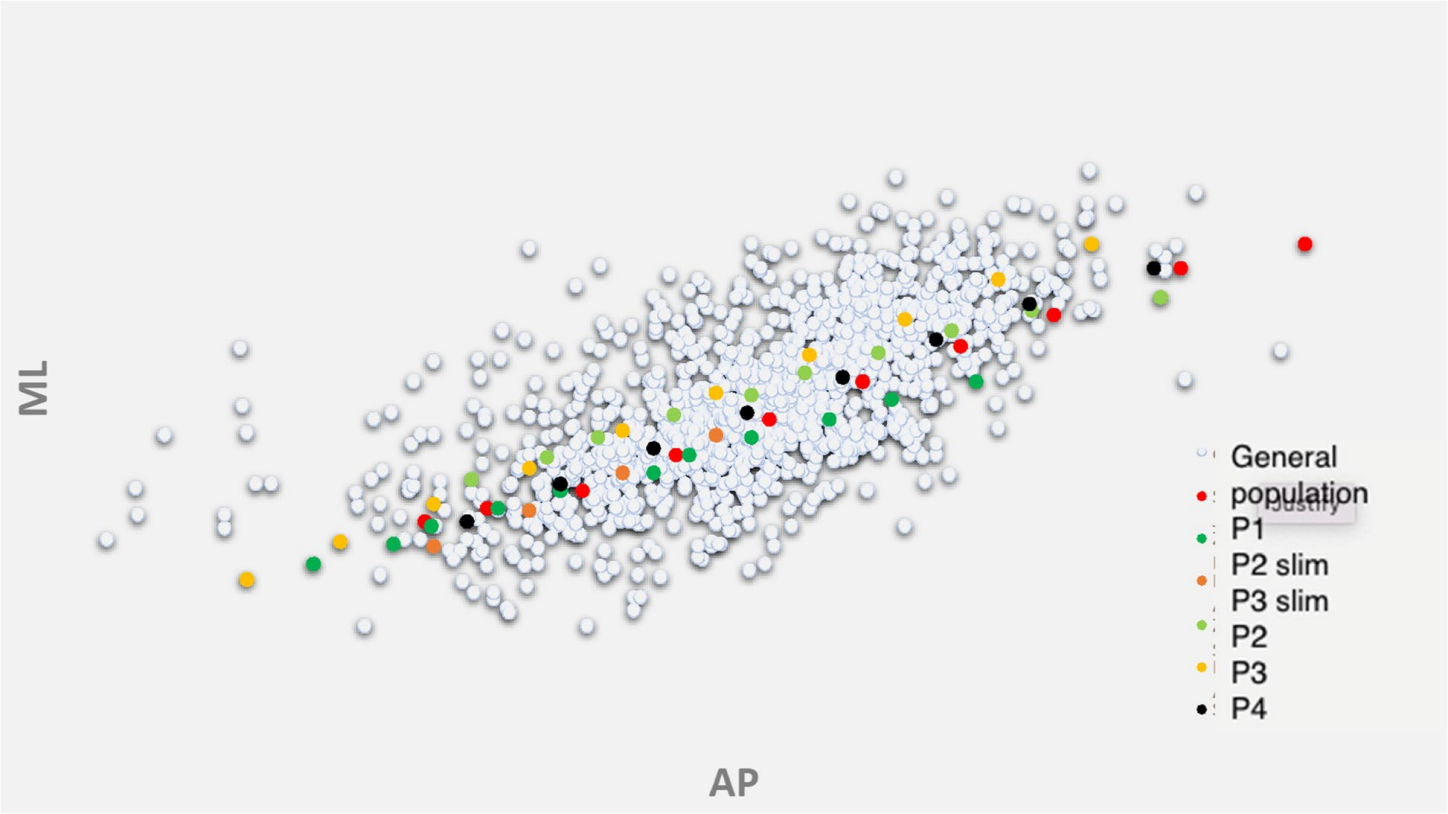
Coverage offered by off the shelf traditional prostheses. Legend: ML medio-lateral, AP: antero-posterior. Each grey dot depicts a femur and each colored dote an implant size

Then, a superimposition in their respective femoral sizing systems of the contemporary OTS knee prostheses femoral sizes on the same graph was performed and an estimate of their fitting is presented in separated graphs for each type of implant in Fig. 5 and altogether in Fig. 6.

**Fig. 7:**
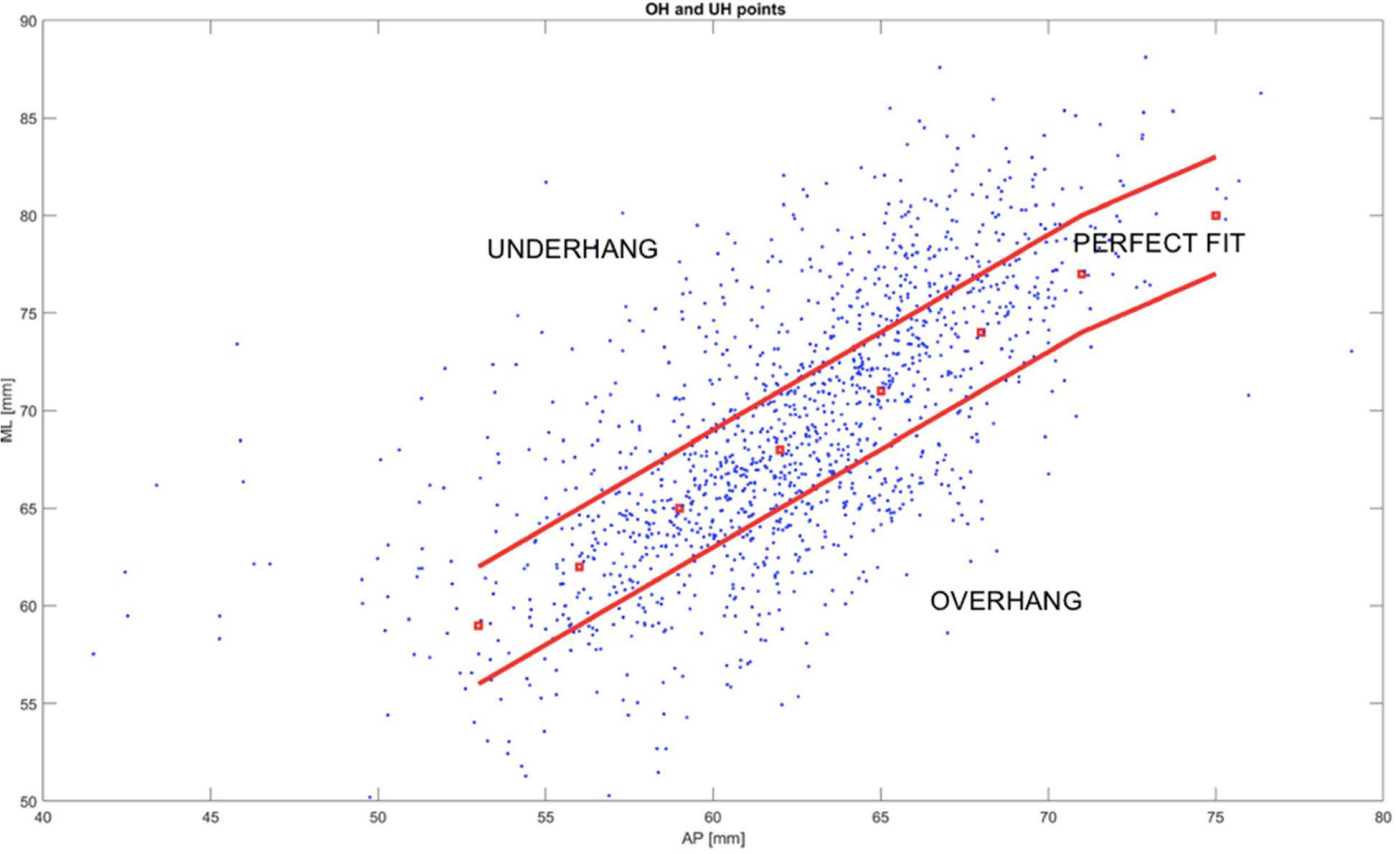
Subadequate fitting of the current OTS femoral component sizes on the ML axis. The red dots represent the femoral system, named “P1” sizes, each dot represents one patient femur The 2 red lines were drawn at 3 mm from each size, for a given femoral AP dimension. The dots within the red lines are the one that have an appropriate fit the ones falling out have instead an inadequate fit in ML minus (overhang) or plus (underhang). It is assumed a perfect fit scenario falls for a given AP for a ML tolerance of +—3 mm

Different OTS contemporary TKA designs are here represented, namely: P1, P2, P3, P4, deemed representatives of the “state-of-the-art” off-the-shelf femoral designs. Two of these femoral TKA systems offer standard sizes (std) and narrower ones (n), respectively P3std, P3n, P4std, P4n. Then, the quantification of the degree of overhang or underhang, estimated to be clinically significant whenever exceeding the cut-off value of 3 mm, as demonstrated by [7], is presented in Fig. 7.

**Fig. 8:**
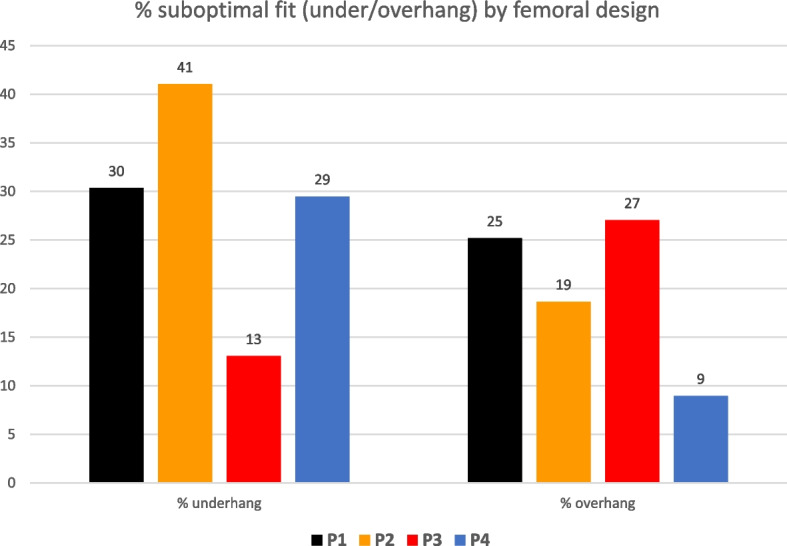
Depicts the percentage of underhang (on the left) and overhang (on the right) for each OTS femoral implant considered in this study

Then, calculation of the percentage of healthy femurs in under- or overhang for each femoral prothesis implant gave rise to an implant-specific fitting performance as presented in Fig. 8.

**Fig. 9:**
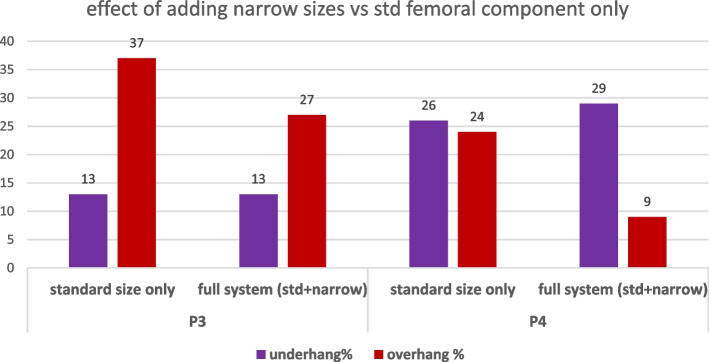
Effect of adding the slim size in the evaluation of underhang and overhang

An additional analysis was performed to evaluate the change in this percentage brought about by the introduction of the narrow implant in two of these OTS contemporary TKA femoral designs at study, namely P3 and P4. For P3 the introduction of narrower sizes P3n vs only having available intra-operatively the P3 std sizes did not solve the underhang but improved (decrement of 10%) the overhang rate above 3 mm. Instead for P4 the introduction of slimmer sizes P4n vs only having the P4 std increased slightly the underhang 3% but decreased the rate of overhang above 3 mm of 15% points as can be visualized in Fig. 9.

## Discussion

The most important finding of the current study is the mismatch between the sizes of the off-the-shelf (OTS) current femoral knee protheses and the actual size of the operated knees, and how this challenge may be addressed by expanding the available sizes of the implants as seen with the introduction of the slim sizes of the femoral systems (P3 and P4). In particular, the anthropometric analysis of 1,395 distal femurs revealed that by employing the available sizes of the contemporary “state-of-the-art” OTS femoral implants up to 13–41% (based on the implant model) would show underhang and 9–27% would give overhang (based on the implant model). And even when some companies released slim sizes thought to be dedicated to women or slimmer femoral morphologies such as Asians, these percentages did not improve the underhang rate and were able to reduce the underhang rate only partially, therefore not representing a substantial solution to assist intra-operative surgeon decision making in femoral size selection.

**Fig. 10:**
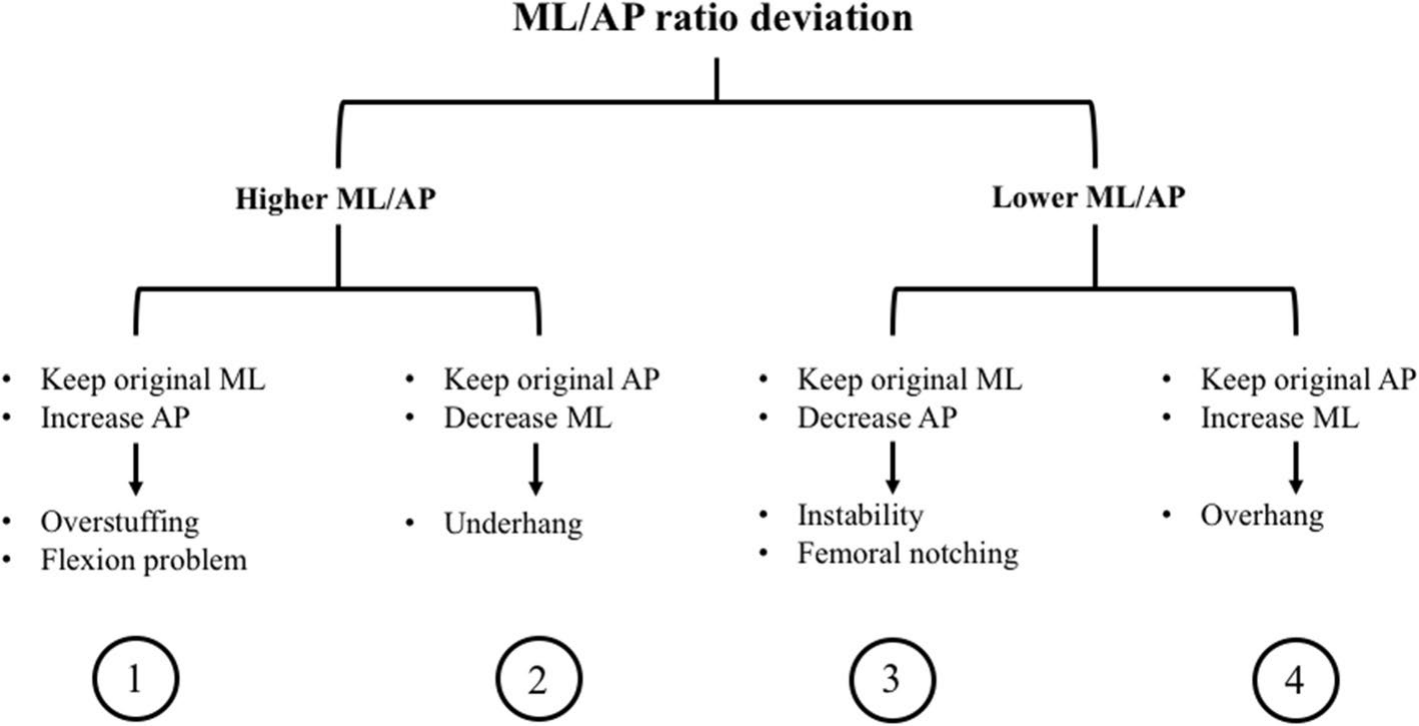
Presents the four possible approaches whenever a ML/AP ratio deviation in encountered during a TKA surgery

As seen in Fig. 6, the commercially available femoral implants are designed for a precise ML/AP ratio (namely “femoral AR Aspect Ratio”) of the femur, and often not sensible to AP variation being approximately constant across different AP, so that for a specific AP size one and only one ML size is available, and the other way around. Therefore, whenever a patient femur deviates from this AR ratio, the surgeon in the operating room faces some technical challenges that can be addressed employing four different approaches depicted in the Fig. 10.

In this case there are two possibilities, either the patient has a higher ML/AP ratio or lower with respect to the available implant offering. In the former case, the surgeon can choose to either keep the patient natural ML and increasing the AP size (approach 1) by resecting less on the AP axis or to keep the patient natural AP and decrease the ML size (approach 2) by resecting more on the ML axis. Unfortunately, these compromises are associated with specific complications. In particular, when approach 1 is chosen, if the surgeon employs a posterior referencing method for the osteotomy it may bring about overstuffing; while instead if he uses an anterior or mixed referencing method, will increase the posterior condylar offset (PCO). In the first case, overstuffing is a known complication of TKA in the literature, and has been associated to decreased passive knee flexion and altered patellar kinematics during knee flexion [5]. On the other hand, a higher PCO is known to predispose to direct impingement of the tibial insert posteriorly against the femur leading to reduced ROM in flexion [1]. This biomechanical consequence is of vital importance for the patient since a minimum of 90° of flexion is required for daily living [13]. In addition, a recent biomechanical study also showed that an increase of just 3 mm in PCO resulted in an increase in contact stress on the polyethylene of 14% during flexion [12]. On the other hand, in case of higher ML/AP ratio the surgeon can keep the patient natural AP and decrease the ML size by resecting more on the ML axis, leading to underhang (approach 2). In turn underhang can possibly lead to medialization of the trochlea [24].

Instead in the latter case, when the patient has a lower ML/AP ratio, the surgeon can choose to either keep the patient natural ML and decrease the AP size (approach 3) by resecting more on the AP axis or to keep the patient natural AP and increasing the ML size (approach 4) by resecting less on the ML axis. Unfortunately, also these compromises are associated with specific complications. When approach 3 is chosen, by resecting more on the AP axis, if the surgeon employs a posterior referencing method for the osteotomy it may bring about femoral notching, while instead if he uses an anterior or mixed referencing method, will decrease the posterior condylar offset ratio (PCOR). Both these outcomes can have a negative impact on the success of the surgery. Indeed, femoral notching is a well-known consequence of an exaggerated width of the AP osteotomy, and has been found to contribute to the risk of periprosthetic fractures after TKA by 17 times [27]. On the other side, if the surgeon employs an anterior referencing method, he will reduce the PCOR, and an excessive reduction of the latter has been associated to instability in flexion [24].

Finally, when approach 4 is chosen, to keep the same AP length the surgeon needs to resect less on the ML axis leading to lateral and/or medial overhang of the implant. Unfortunately, overhanging, defined as a discrepancy of ≥ 3 mm between the implant and the original knee, was correlated with a twofold increased risk of knee pain at 2 yeas of follow up [17] being clinically-relevant, and it was demonstrated to be associated to increased pain [9] poorer ROM and generally inferior PROMs, including limited function.

To sum up, whenever a ML/AP mismatch in encountered adaptations are needed and these bring about deleterious biomechanical and clinical complications. This concept becomes of particular relevance in light of the fact that ML/AP mismatch is rather frequent in men (40%) and even more in women (68%) [17]. The reason behind the large spread of this poor fit is that commercially available implants come in a limited range concerning the ML/AP ratio.

Indeed, Dai et al. [9] found that the best fit were present for implants design with multiple ML offerings per AP size, since they provide more sizes selections to match the different ML sizes of the distal femur, compared to designs with single ML offerings.

Femoral component mismatch has been observed not only to be frequent, as high as 87%, but also to negatively correlate with outcomes at 2-years of follow up [25].

The abovementioned strategies to overcome AP-ML mismatch belong to the traditional orthopedic surgeon, however nowadays digital simulations on the patient’s knee CT scan allow predicting the bony coverage of the component and to adjust the cuts and the component size accordingly [22].

The limitations of the current study are represented by the retrospective nature of the study, the fact that only cruciate retaining (CR) versions of the implants were analyzed and by the nature of AP measurement. Indeed, different articles adopted different definitions for the AP dimension, sometimes being based on a medial aspect, or lateral one or more frequently an averaged mean of the two values. The latter was finally adopted in the ML vs AP anthropometric diagrams but excluding all the other studies that measure the AP on the medial and lateral side may have acted as a source of bias. The fact that only healthy femurs were included in the database represents a limitation since femurs undergoing TKA by definitions are not healthy but typically present signs of OA which translates directly in affected morphologies by the pathological changes. Another potential source of bias is represented by the paucity of data to determine the threshold for over – and underhang, indeed the current study adopts a cut-off of 3 mm above (overhang) or below (underhang) but this setting was based only on one study [15], and future studies may set another threshold to determine clinically significant over- or underhang. The current study focused on the suboptimal femoral ML fit in the general population, but future studies should address this inadequacy on a gender and racial bases as distinct covariates since anthropomorphic studies have showed the existence of morphologic differences based on ethnicity, in studies not just based on traditionally Caucasian knee [14, 23, 26].

## Conclusions

Even contemporary carefully designed and highly engineered recently-introduced OTS TKA systems fail to limit significantly or avoid at all the femoral suboptimal fit rate above clinically relevant cut-off, such as over/underhang with all the clinical consequence of potential ML soft tissue irritation, unexplained residual pain, limited function (ROM), or conversely exposed resected bony surface. The introduction of the slim versions of the current OTS Knee protheses has brought about improvements in the protheses fitting but still the adequacy of fit remains insufficient.

## Data Availability

Stored on data repository.
