# Correction: Sub‑optimal femoral fit in total knee arthroplasty, a systematic review of human femoral data vs off‑the‑shelf contemporary femoral components

**DOI:** 10.1186/s40634-023-00619-7

**Published:** 2023-05-25

**Authors:** Tommaso Bonanzinga, Francesco Manlio Gambaro, Francesco Iacono, Maurilio Marcacci

**Affiliations:** 1 IRCCS Humanitas Research Hospital, Via Manzoni 56, 20089 Rozzano, Milan Italy; 2 Department of Biomedical Sciences, Humanitas University, Via Rita Levi Montalcini 4, 20090 Pieve Emanuele, Milan Italy

**Correction: J Exp Ortop 10, 41 (2023)**

10.1186/s40634-023-00607-x

Following publication of the original article [1], the authors’ identified errors on affiliations 1 and 2.

The correct affiliations are shown in this article and the original article [1] has been corrected.

## References

[1] Bonanzinga T, Gambaro FM, Iacono F (2023). Sub-optimal femoral fit in total knee arthroplasty, a systematic review of human femoral data vs off-the-shelf contemporary femoral components. J Exp Ortop.

